# Correction: Diverse microtubule-targeted anticancer agents kill cells by inducing chromosome missegregation on multipolar spindles

**DOI:** 10.1371/journal.pbio.3003039

**Published:** 2025-02-12

**Authors:** Amber S. Zhou, John B. Tucker, Christina M. Scribano, Andrew R. Lynch, Caleb L. Carlsen, Sophia T. Pop-Vicas, Srishrika M. Pattaswamy, Mark E. Burkard, Beth A. Weaver

## Notice of Republication

Incorrect versions of Fig 1 and S1 Data were published in error. This article was republished on January 24, 2025 to correct this error. Please download this article again to view the correct version.

**Fig 1:**
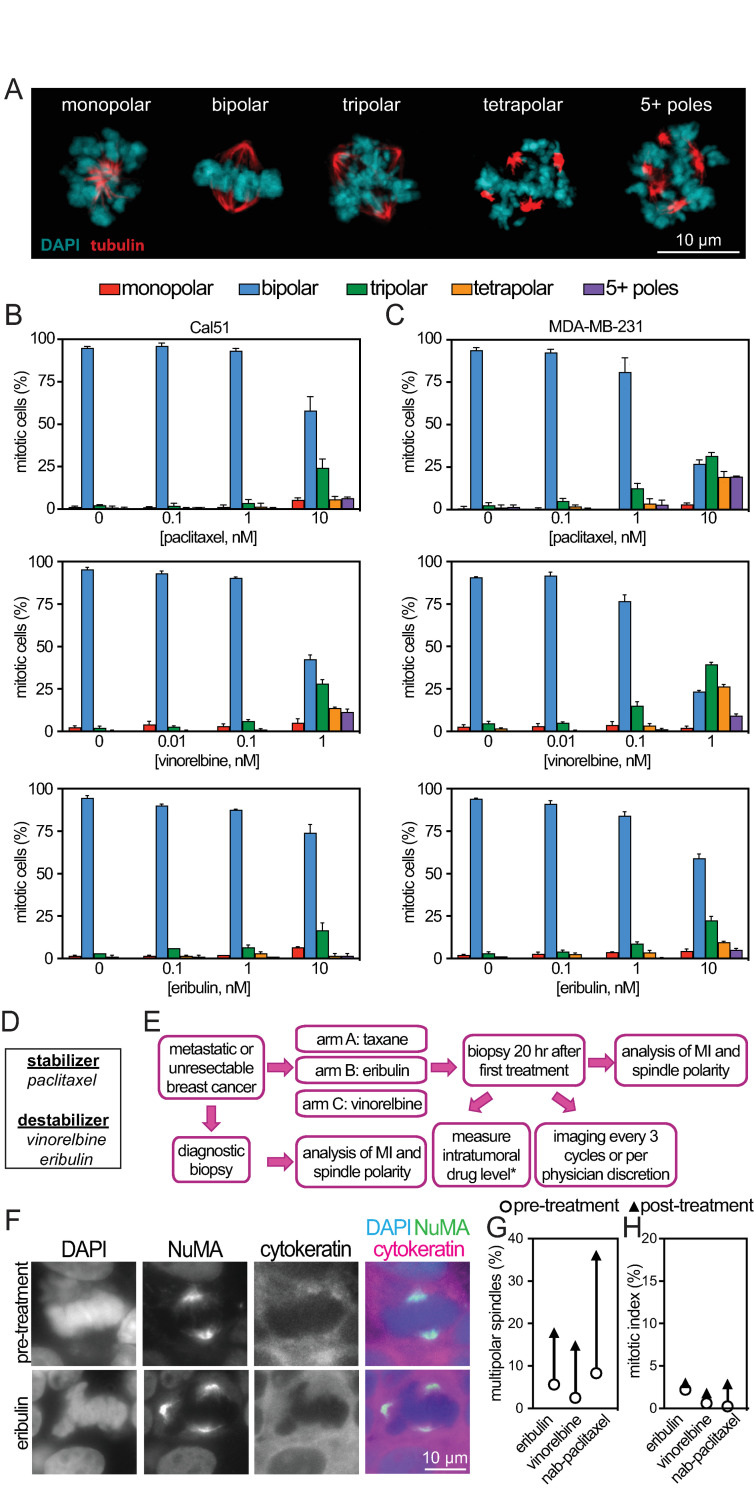


## Supporting information

S1 Data (XLSX)
