# Supplementary material for: Diverse microtubule-targeted anticancer agents kill cells by inducing chromosome missegregation on multipolar spindles
Source: PLoS Biol. 2023 Oct 26;21(10):e3002339. doi: 10.1371/journal.pbio.3002339 (PMC10602348; doi:10.1371/journal.pbio.3002339)
Supplement: S1 File — Fig A. Multipolar spindle induction is a conserved mechanism of clinically useful microtubule poisons. Diverse microtubule poisons are capable of inducing multipolar spindles at low nM doses in (A) Cal51 and (B) MDA-MB-231 breast cancer cells after 20 hours of treatment. n ≥ 100 cells in each of 3 biological replicates. Data used to generate graphs can be found in S1 Data. Fig B. Low concentrations of microtubule poisons do not induce mitotic arrest. Mitotic index after 20 hours of treatment with the indicated concentrations of microtubule poisons in (A) Cal51 and (B) MDA-MB-231 cells. n ≥ 295 cells across 3 independent replicates. Data used to generate graphs can be found in S1 Data. Fig C. The clinically ineffective microtubule poisons nocodazole and colcemid do not induce multipolar spindles. Cal51 and MDA-MB-231 cells were treated with the indicated concentrations of nocodazole (A-D) or colcemid (E-H) for 20 hours and scored for spindle multipolarity (A, B, E, F) and mitotic index (C, D, G, H). n = 100 cells for spindle polarity and 250 cells for mitotic index in each of 3 independent replicates. Data used to generate graphs can be found in S1 Data. Fig D. Validation of CIN-inducible cell lines. (A, B) Representative images of interphase cells with (A) normal and (B) amplified centriole numbers. Images were acquired from Plk4-inducible MCF10A cells treated with (A) water or (B) 2 μg/mL dox for 72 hours. (C, D) Quantitation of centriole (C) amplification and (D) number in dox-inducible Plk4 MCF10A cells after 72 hours of dox treatment. n = 100 cells in each of 3 biological replicates. (E) Relative Kif2b expression 72 hours after transfection with indicated shRNA. Since endogenous Kif2b expression is below the lower limit of detection, and fluorescently labeled exogenous Kif2b has previously been shown to be a suitable surrogate for endogenous Kif2b [107,108], validation of Kif2b depletion was performed in HEK293T cells stably expressing Kif2b-mNeonGreen. Kif2b ex [file pbio.3002339.s001.pdf]

S1 File: Supplementary Figures and Tables

Diverse microtubule-targeted anticancer agents kill cells by inducing chromosome missegregation on multipolar spindles

Amber S. Zhou[1], John B. Tucker[2], Christina M. Scribano[1], Andrew R. Lynch[3], Caleb L. Carlsen[4], Sophia T. Pop-Vicas[5], Srishrika M. Pattaswamy[5], Mark E. Burkard[6,7,8], Beth A. Weaver[5,7,8,*]

[1]Molecular and Cellular Pharmacology Graduate Training Program, University of Wisconsin, Madison, Wisconsin, USA

[2]Cancer Biology Graduate Training Program, University of Wisconsin, Madison, Wisconsin, USA

[3]Cellular and Molecular Pathology Graduate Training Program, University of Wisconsin, Madison, Wisconsin, USA

[4]Cellular and Molecular Biology Graduate Training Program, University of Wisconsin, Madison, Wisconsin, USA

[5]Department of Cell and Regenerative Biology, University of Wisconsin, Madison, Wisconsin, USA

[6]Department of Medicine, University of Wisconsin, Madison, Wisconsin, USA

[7]Department of Oncology/McArdle Laboratory for Cancer Research, University of Wisconsin, Madison, Wisconsin, USA

[8]Carbone Cancer Center, University of Wisconsin, Madison, Wisconsin, USA

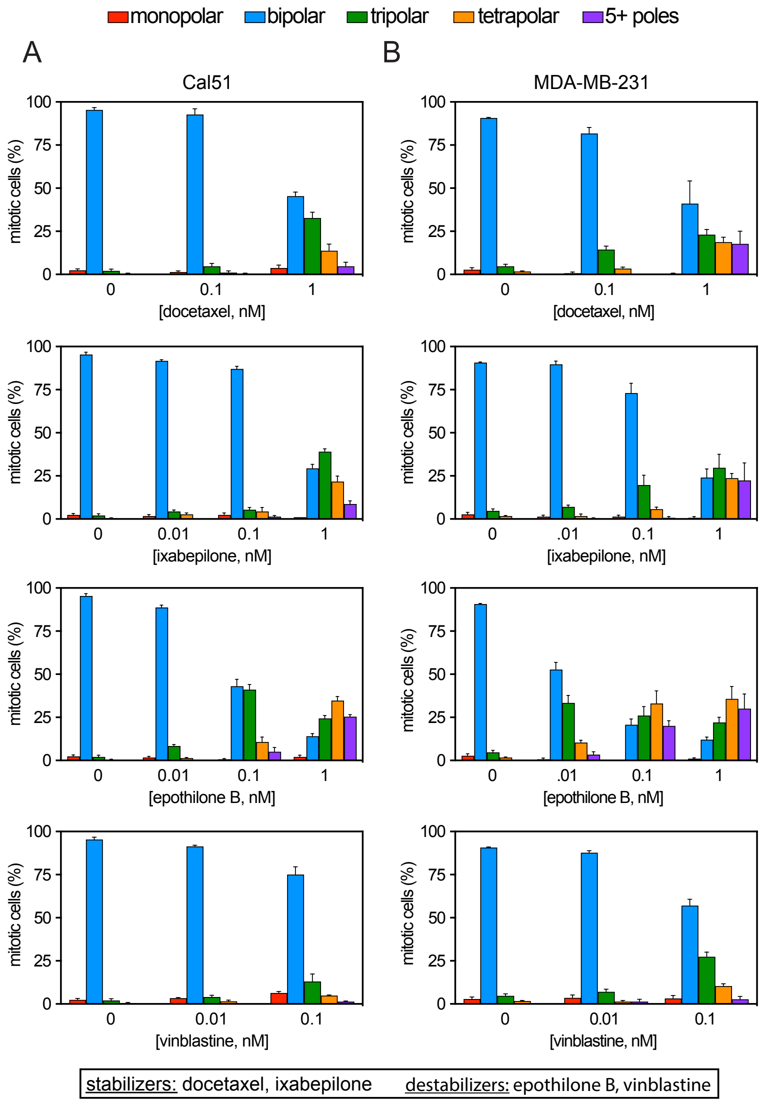

Supporting Fig A. Multipolar spindle induction is a conserved mechanism of clinically useful microtubule poisons. Diverse microtubule poisons are capable of inducing multipolar spindles at low nM doses in A) Cal51 and B) MDA-MB-231 breast cancer cells after 20 hours of treatment. n≥100 cells in each of 3 biological replicates. Data used to generate graphs can be found in S1 Data.

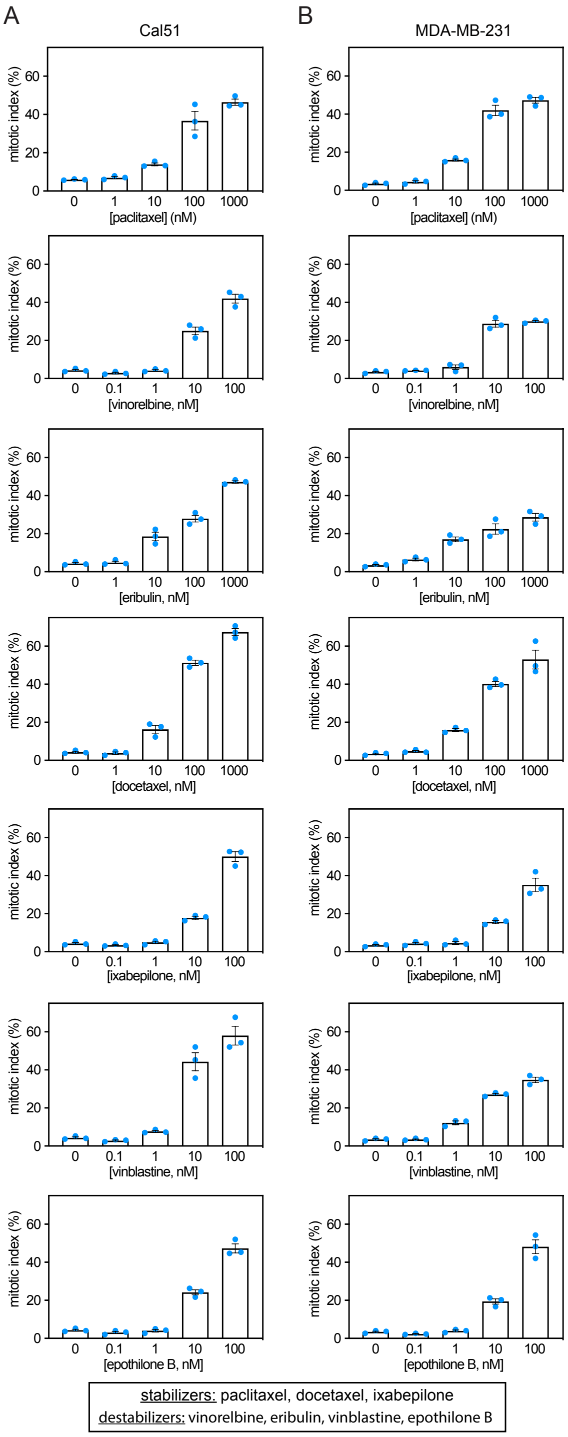

Supporting Fig B. Low concentrations of microtubule poisons do not induce mitotic arrest. Mitotic index after 20 hours of treatment with the indicated concentrations of microtubule poisons in A) Cal51 and B) MDA-MB-231 cells. n≥295 cells across 3 independent replicates. Data used to generate graphs can be found in S1 Data.

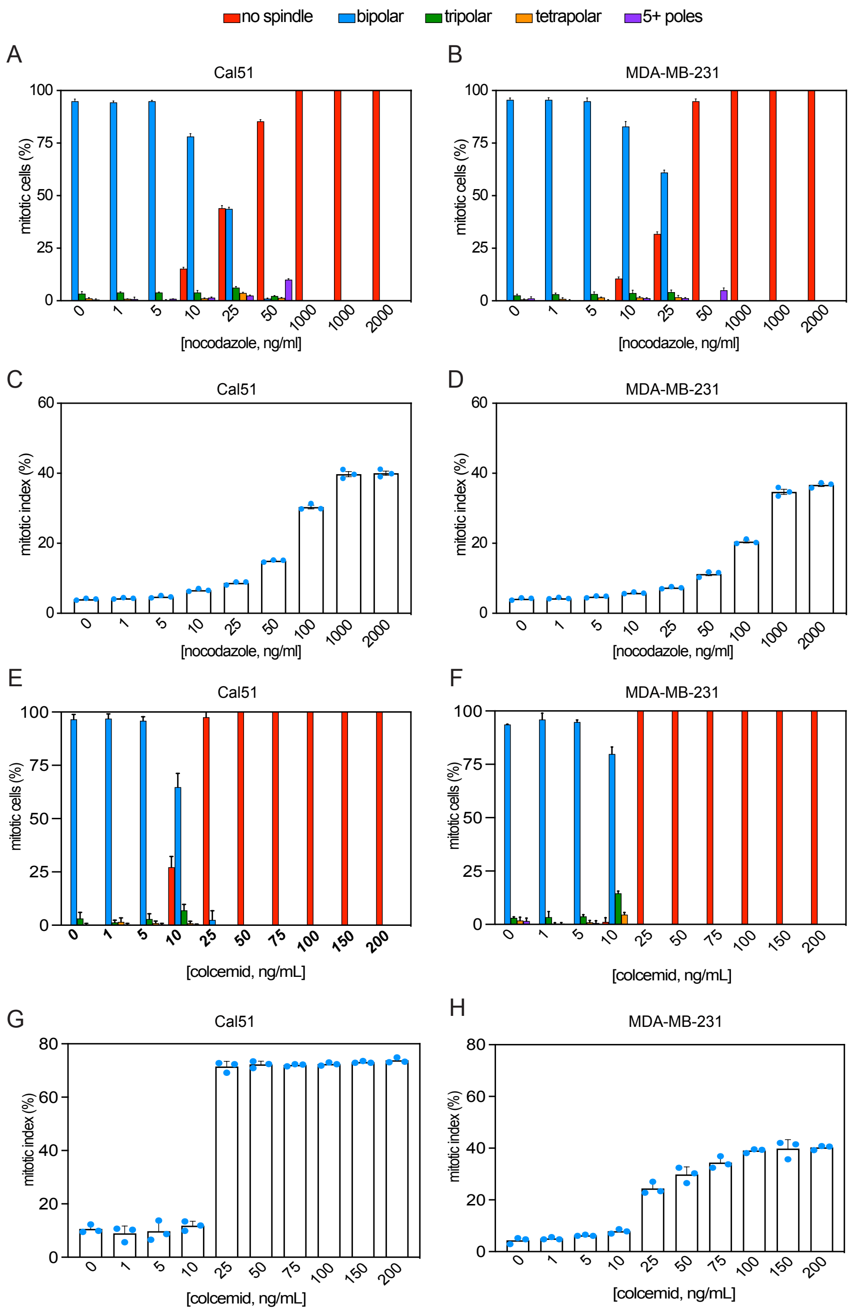

Supporting Fig C. The clinically ineffective microtubule poisons nocodazole and colcemid do not induce multipolar spindles. Cal51 and MDA-MB-231 cells were treated with the indicated concentrations of nocodazole (A-D) or colcemid (E-H) for 20 hours and scored for spindle multipolarity (A-B, E-F) and mitotic index (C-D, G-H). n=100 cells for spindle polarity and 250 cells for mitotic index in each of 3 independent replicates. Data used to generate graphs can be found in S1 Data.

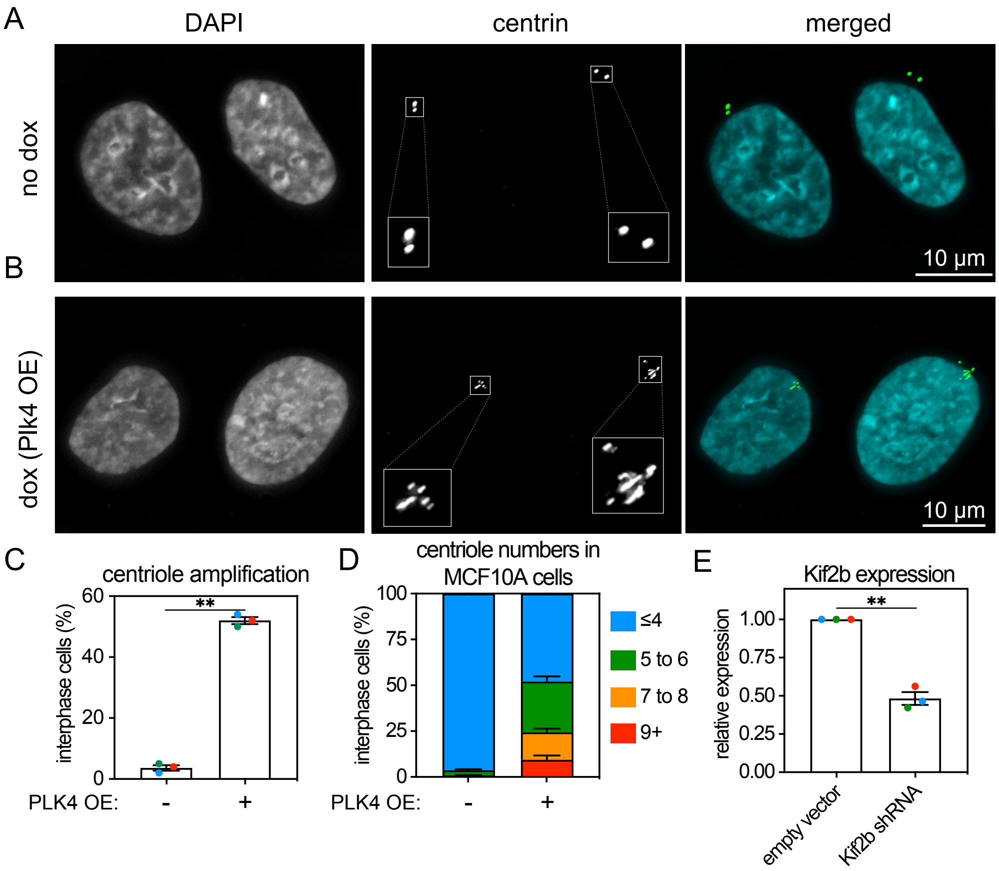

Supporting Fig D. Validation of CIN-inducible cell lines. A-B) Representative images of interphase cells with (A) normal and (B) amplified centriole numbers. Images were acquired from Plk4-inducible MCF10A cells treated with (A) water or (B) 2 μg/mL dox for 72 hours. C-D) Quantitation of centriole (C) amplification and (D) number in dox-inducible Plk4 MCF10A cells after 72 hours of dox treatment. n=100 cells in each of 3 biological replicates. E) Relative Kif2b expression 72 hours after transfection with indicated shRNA. Since endogenous Kif2b expression is below the lower limit of detection, and fluorescently labeled exogenous Kif2b has previously been shown to be a suitable surrogate for endogenous Kif2b [107, 108], validation of Kif2b depletion was performed in HEK293T cells stably expressing Kif2b-mNeonGreen. Kif2b expression was normalized to GAPDH expression and empty vector. n = 3 biological replicates. Unpaired t-test was performed to determine statistical significance. Data used to generate graphs can be found in S1 Data. ** indicates p<0.01.

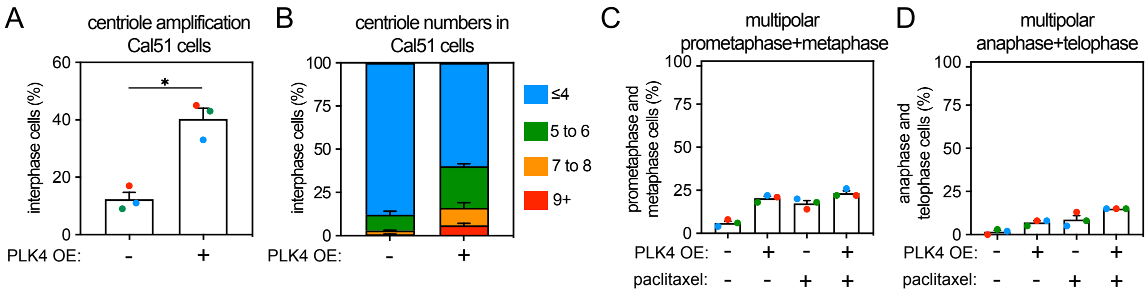

Supporting Fig E. Centriole amplification does not induce multipolar divisions in Cal51 breast cancer cells, which are proficient at focusing multipolar spindles. A-B) Quantitation of centriole A) amplification and B) number in dox-inducible Plk4 Cal51 cells after 48 hours dox treatment showing dox inducible centriole amplification. n=100 cells in each of 3 biological replicates. C-D) Quantification of multipolar spindles in C) early stages of mitosis (prometaphase and metaphase) and D) late stages of mitosis (anaphase and telophase) in dox-inducible Plk4 Cal51 cells, showing centriole amplification induces a much lower rate of multipolar spindles in Cal51 cells than in MCF10A cells, even in the presence of subclinical paclitaxel (compare to figures 2A, 2B, and S4C). n=100 cells in each of 3 biological replicates. Unpaired t-test was performed to determine statistical significance. Data used to generate graphs can be found in S1 Data. * indicates $p<0.05$.

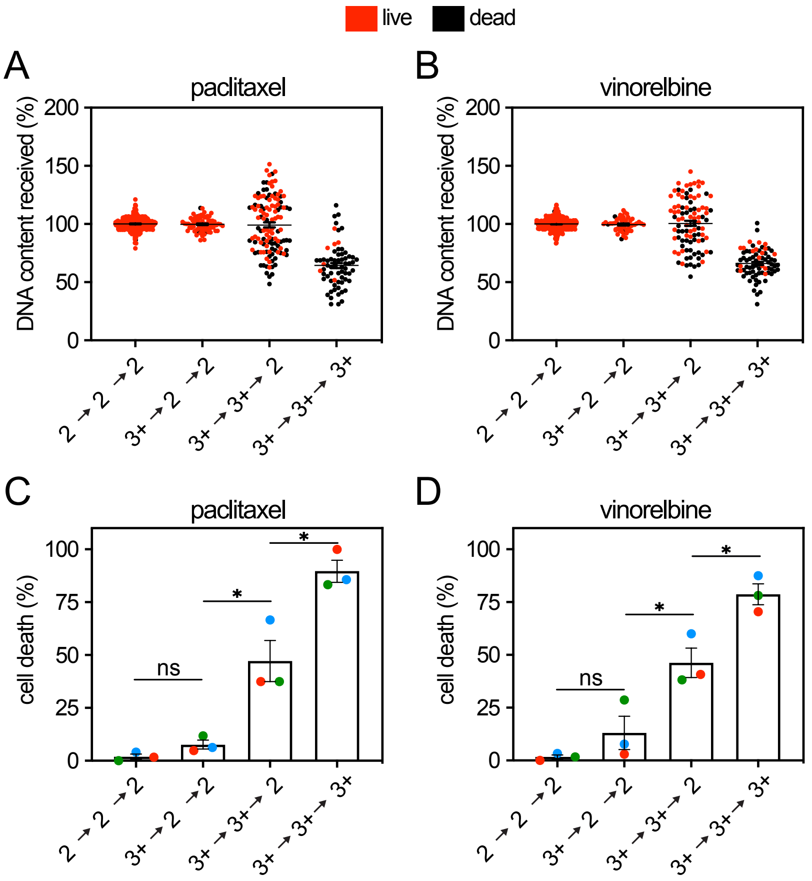

Supporting Fig F. Sustained multipolarity in the presence of microtubule poisons corresponds with cell death. 72 hour timelapse imaging of Plk4-inducible MCF10A cells stably expressing histone H2B-mNeonGreen and mScarlet-tubulin treated with 1 nM paclitaxel or 1 nM vinorelbine +/- 2 µg/mL dox to induce Plk4. A-B) Quantification of DNA content in daughter cells at early G1 categorized by type of division showing that multipolar divisions increase DNA loss and cell death. n≥50 cells per replicate in each of 3 biological replicates. C-D) Quantification of cell death after the specified type of division showing that persistent multipolar (3^{+}->3^{+}->3^{+}) divisions are the most lethal across both C) paclitaxel and D) vinorelbine. Color represents specific replicate, and bars represent mean +/- SEM. n ≥ 40 cells per category across 3 biological replicates. Unpaired t-test was performed to determine statistical significance. Data used to generate graphs can be found in S1 Data. * indicates p<0.05, ns indicates not significant.

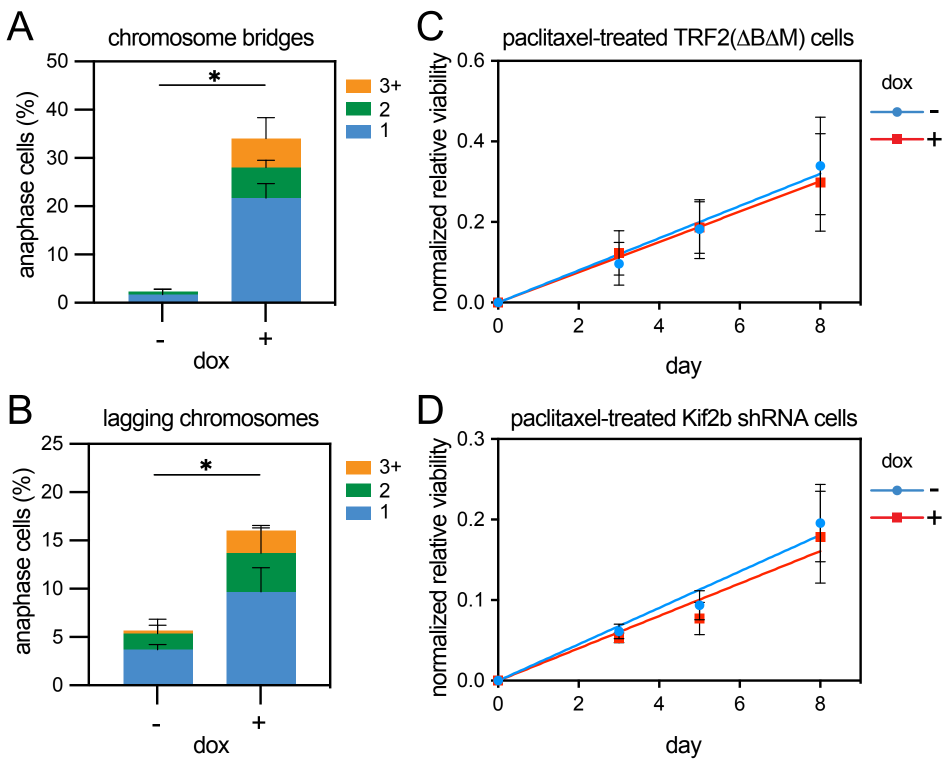

Supporting Fig G. A single missegregated chromosome is not sufficient to sensitize to paclitaxel. A) Quantitation of chromosome bridges in dox-inducible TRF2(ΔBΔM)-mScarlet Cal51 cells after 72 hour dox treatment. n=50 cells in each of 3 biological replicates. B) Quantitation of lagging chromosomes in dox-inducible Kif2b shRNA Cal51 cells treated with dox for 72 hours. n=50 cells in each of 3 biological replicates. C-D) Dox treatment of dox-inducible TRF2(ΔBΔM)-mScarlet (C) or Kif2b shRNA (D) Cal51 cells does not increase sensitivity to paclitaxel. Cells were treated with 2 μg/mL dox for 48 hours before treatment with 2.5 nM paclitaxel for the indicated number of days. n = 3 biological replicates. Unpaired t-test was performed to determine statistical significance. Data used to generate graphs can be found in S1 Data. * indicates $p<0.05$.

Patient	Drug	Site of biopsy	Subtype	Age	Race	Ethnicity	Response
1	Eribulin	Liver	ER+/PR+/HER2-	59	White	Non-Hispanic or Latino	N/A
2	Vinorelbine	Peritoneum	ER+/PR-/HER2-	62	White	Non-Hispanic or Latino	Progression
3	Nab-paclitaxel	Liver	ER+/PR+/HER2-	69	White	Non-Hispanic or Latino	Stable Disease

Table A. Metastatic microtubule poison trial patient characteristics. ER: Estrogen Receptor. PR: Progesterone Receptor. HER2: human epidermal growth factor receptor 2. All patients in this study had metastatic spread. Patient response was determined by review of imaging reports and RECIST 1.1 criteria [106]. Response information was not available for Patient 1 because they only received one dose of eribulin.

	[Eribulin, nM]	Degree of concentration
Plasma	1.3	NA
Tumor core 1	57.7	46x
Tumor core 2	41.1	33x

Table B. Intratumoral eribulin concentration. Eribulin concentration was measured by HPLC analysis 20 hours after the first dose of eribulin in plasma and two tumor cores from a single patient. Eribulin was quantified assuming a tumor density of $1g/cm^3$. NA=not applicable.

Compound 1 [vinorelbine, nM]	Compound 2 [GSK923295, nM]	Fraction Affected	Parameters			Combination Index
			m	Dm	r	
5	0	0.75	0.39663 +/- 0.11910	0.11618	0.92045	NA
10	0	0.87				NA
20	0	0.92				NA
500	0	0.96				NA
0	50	0.20	4.47445 +/- 0.56801	64.4316	0.98426	NA
0	60	0.47				NA
0	70	0.64				NA
0	100	0.86				NA
5	70	0.91	NA	NA	NA	0.77384
10	70	0.92				0.81165
20	70	0.95				0.66538
500	70	0.96				1.95967

Table C. CENP-E inhibition is synergistic with low doses of vinorelbine. Chou-Talalay non-constant ratio synergy testing of vinorelbine with the CENP-E inhibitor GSK923295 in Cal51 cells. Combination Index (CI)=1 indicates an additive response, CI>1 an antagonistic one, and CI<1 synergistic. m = kinetic order of single drug curves, Dm = IC_{50}, r = linear correlation coefficient for median affect plot, NA=not applicable.
