# Supplementary material for: Diverse microtubule-targeted anticancer agents kill cells by inducing chromosome missegregation on multipolar spindles
Source: PLoS Biol. 2023 Oct 26;21(10):e3002339. doi: 10.1371/journal.pbio.3002339 (PMC10602348; doi:10.1371/journal.pbio.3002339)
Supplement: Attachment — Submitted filename: PLoS Biology Reviewer Comments and Rebuttal 2023-08-30.pdf [file pbio.3002339.s007.pdf]

Reviewer 1 (Claire E Walczak, signs review):
Anti-microtubule agent are powerful therapeutics used as front-line treatments in a number of cancers. They have been historically thought to act by arresting cells in mitosis, leading to apoptosis. However, groundbreaking work by the Weaver lab showed that in breast cancer, paclitaxel did not induce mitotic arrest, but rather cells had to pass through mitosis leading to chromosomal instability (CIN), aneuploidy and the formation of micronuclei. This finding suggests that drugs that halt the cell cycle may not be therapeutically valuable, but instead that targeting CIN may be the key mechanism of action.

In the present study, the authors show that other clinically used anti-microtubule agents also generate CIN, leading to cell death as a conserved mechanism of action. Generation of CIN was only observed in the anti-microtubule agents that were used clinically and did not correlate with microtubule-targeting drugs, such as nocodazole, that depolymerize microtubules and are not used therapeutically. They also develop a new method to quantify CIN, which will be a valuable addition to the field.

Overall, the findings in this paper are highly significant both for the cell biology community as well as the cancer biology field, as it is paradigm shifting in thinking about the mechanistic action of anti-microtubule agents. The data is strong and convincing of the points made. I have a few small suggestions for improvement of the paper before publication.

We thank Dr. Walczak for her careful evaluation of our work and its impact in the fields of cancer biology and cell biology.

1. *Throughout the manuscript, the authors need to clearly specify the number of samples analyzed in terms of patient samples, cells, number of replicates and which statistical tests were used.*

Thank you for raising this point. The manuscript has been revised to clearly specify this information, including detailing patient information in the legend for Figure 1 and new Supplemental Table 1, adding the number of cells and replicates to the legends for Figures 2 and 3, and specifying the statistical tests used in the legends for Figures 2, 4-6 and S4-7.

2. *There are no controls to validate the overexpression of Plk4 or the knockdown of Kif2B.*

Good point. We had performed these validations when beginning the experiments but realize now we neglected to include them in the original version of the manuscript. Validation of Plk4 overexpression and Kib2B knockdown is shown in the new Supplemental Figure 4A-E.

3. *In the data in Figure 3, they clearly show that the assay can detect a single chromosome mis-segregation event, but I am curious if the assay is sensitive enough to detect loss of a chromosome in the DNA mass. Also- in Figure 3D, five chromosome pairs is an insufficient number of samples to analyze. If the authors did three independent repeats, that is only one to two chromosome pairs per experiment.*

We were also curious whether the assay is sufficiently sensitive to detect loss of a single chromosome in a DNA mass and attempted to test this using a U2OS cell line in which chromosome 1 can be visualized due to incorporation of 256 copies of the lac operator sequence in chromosome 1 coupled with expression of YFP-tagged lac repressor (Janicki S. et al., *Cell,* 2004). We planned to quantify DNA content in segregating masses of DNA in which both copies of chromosome 1 segregated to the same daughter cell. Unfortunately, these cells did not proliferate sufficiently in our hands to perform these experiments, despite successful culturing of parental U2OS cells. Thus, this attempt was unsuccessful and we indicate that this is still an open question in the revised discussion on page 17. We have now included an additional 25 chromosome

pairs, for a total of 30 pairs across 3 biological replicates, to validate that calculated DNA content is stable over time in Figure 3D.

4. In Figure 4E, it would be helpful if the authors chose a different color scheme for the Kaplan Meier curves; they are difficult to read.

Thanks for bringing this to our attention. We have changed the color scheme in Figure 4E (now Figure 4F).

5. I am confused by the data using the Cenp-E inhibitor, my recollection is that Cenp-E inhibition leads to a sustained mitotic arrest before failure in checkpoint inhibition.

Yes, high concentrations of CENP-E inhibitor often cause mitotic arrest. For these experiments we used a lower concentration that allows most cells to proceed into anaphase, even though some have polar chromosomes. We have now clarified this in the text on page 14.

Reviewer 2:

The paper provides an interesting incremental advance to the field's understanding of microtubule poisons, in particular demonstrating a common mechanism of action across microtubule stabilisers and destabilisers in inducing CIN via multipolarity. They also provide a useful methodology for quantifying CIN resulting from multipolarity, and use it to demonstrate that DNA loss is most detrimental to the cells in their study. In agreement with their previous work, they find that spindle refocussing reduces CIN, providing resistance to microtubule poisons, and that refocussing can be driven by asymmetrical spread of DNA across the poles. Finally, they find that using a second drug to drive misalignment can result in daughter cell death despite spindle refocussing. Overall, the study is well done suitable for publication. Below are a number of minor comments designed to help improve the presentation for the reader.

We thank the Reviewer for their positive comments and suggestions for improving the manuscript.

Figure 1
Need more clinical information re clinical study: How many patients were included in the study? How many received each treatment and how many patients contributed to Fig.1 panels F and G (the legend currently only gives numbers of cells, are these from one or multiple patient samples?)
Not sure what they mean by a "clinical touchstone?"
Fig1F in the key the triangle is labelled "C1D2". No clear. Label as "post-treatment"

We thank the Reviewer for these opportunities to clarify the manuscript. We have clarified the legend for Figure 1 and added detailed patient information in the new Supplemental Table 1. We have changed the phrase “clinical touchstone” to “clinical benchmark” and “C1D2” to “post-treatment” in Figure 1F-G (now Figure 1G-H).

Figure 2
Legend states "prometaphase" but text states "prometaphase and metaphase".
We have changed the y-axis and legend to say “prometaphase and metaphase”.

Not clear if comparisons without stats are non-significant i.e. is the increase in multipolarity with PLK4 induction alone in A/B non-significant? Later plots do use "NS" to label non-significant comparisons (maybe should be consistent).
Why are the stats comparisons across different treatments missing from A (but included in B and C)?

We initially did not include certain statistical comparisons out of concern of making the graph too busy. However, we see that this led to confusion and have now included the same statistical comparisons for Figure 2A, B, and C as well as adding “ns” when appropriate to additional figure panels to keep the presentation consistent.

Consider discussing the increase in multipolar spindles with PLK4 induction alone (Panels A, B and E) and how this contributes to the impact of the microtubule poisons + PLK4 OE (i.e. do the combinations have significantly more multipolarity than PLK4 OE alone early in mitosis or just late mitosis).

The combinations do not have significantly more multipolar spindles early in mitosis. This effect is observed only late in mitosis. This is now clarified in the text (page 7) and by the additional statistical comparisons in Figure 2A described in the previous point.

F and G: The frequency of death for 2,2,2 divisions does not seem consistent between these two panels. Are the data for 2,2,2 and 3,2,2 switched in Panel G?

The reason the frequency of death is not identical in these panels is that Figure 2F includes data from cells in all 6 treatment groups (including DMSO) combined whereas Figure 2G shows data from only 2 treatment groups – Plk4+paclitaxel and Plk4+vinorelbine – and these are shown individually. This has now been clarified in the figure legend.

H-I: Range of number of cells counted is quite large (22-246); could break this down by condition to allow the reader to understand how many cells contributed to each condition.
Legend doesn't state what the bars on the bar graphs represent.
Legend should state that the colours represent replicates. Or make all replicates black as the colouring of the replicates gets confusing alongside the colouring in panel E.

We have adjusted our figure legend to distinguish the number of cells analyzed for each condition and to clarify what the bars and colors represent. We have also changed the color of replicates in Figure 2A, B, C, F, and G to grayscale to avoid confusion with the colors in panels E, H and I.

Figure 3
Panel C: Not sure why also showing 25 and 50 mins before NEBD? These timepoints are not mentioned in the text.
Legend doesn't say what the bars on the plots represent?
Not clear what cell line is used in C-E.

Our goal in including the 25 and 50 minute time points before NEBD in Figure 3C was to determine whether the timing with which we measured the DNA content in the mother cells was critical or whether this measurement remained consistent over time, as expected. We have clarified this in the text on page 10. In the figure legend we have defined the bars and specified that MCF10A cells were used in panels C-E.

Figure 4
Legend doesn't say what the bars on the plots represent.

We have now defined that the bars represent mean +/- SEM in the figure legend.

Figure 5

Legend and text don't describe the cell line or the treatment used to generate the multipolar divisions for the whole figure.
Panel A: Is it the % of the "multipolar anaphase cells" or the % of all anaphase cells (think the former i.e. of the multipolar they are mostly tripolar)? Should make clearer.
Panel B: Should the y-axis have the same label as the y-axis in panel A?
Legend doesn't say what the bars on the plots represent.

We have included the cell line and treatments used to generate multipolar divisions in the legend for Figure 5. We have also adjusted the axes titles of panels A and B to indicate “multipolar anaphase cells”, as suggested. We also clarified the description of panel B in the text on page 12 and in the legend for Figure 5. Bars represent mean +/- SEM, which is now defined in the figure legend.

Reviewer 3:

In their previous papers, the Weaver lab has shown that low doses of microtubule stabilising drug paclitaxel on cultured cells that mimic clinically relevant doses, apparently cause cell death through promoting multipolar divisions and aneuploidy, rather than the prolonged mitotic arrest seen in higher doses. They also related cell death to the capacity for cancer cells to focus/cluster their multipolar spindles into pseudo-bipolar spindles; the earlier they did this in mitosis, the better their chance of successful cell division and surviving. Using a HSET inhibitor or Plk4 overexpression increased cell death through multipolar divisions, whilst increasing chromosomal instability through a CENPEi synergistically increased cell death with paclitaxel, and inherently high level of CIN in tumours was a good prognosticator for taxane treatment efficacy.

In this paper, Zhou et al have now expanded this work to include both several different stabilisers and also microtubule destabilising drugs. They confirmed that both types of drugs (with the notable exception of nocodazole) alone promote multipolar divisions and cell death, and that this effect is exacerbated with Plk4-related centrosome amplification and again was related to whether cells clustered their multipolar into bipolar spindles early enough in mitosis to avoid a multipolar division. They used histones tagged with fluorescent markers to track DNA content in live cell division, and assessed that cells that tended to lose over 20% of their genome during aberrant divisions were more likely to die and to die faster, than cells that gained 20% extra DNA. Instability generated by Kif2b shRNA or telomere dysfunction which generally resulted in only lagging chromosomes or bridges was insufficient to sensitise to paclitaxel. Furthermore, CENPEi was useful for promoting extra cell death in cell line Cal51 which was resistant to paclitaxel due to effective spindle focusing, by working synergistically.

Overall, major conclusions of their study are that (i) low doses of either microtubule stabilising, or destabilising agents, that are representative of clinically achieved intratumoural levels, induce multipolar spindles in breast cancer cells, (ii) cells can be sensitised to low doses of microtubule disrupting agents by either increasing centrosome numbers (using Plk4 overexpression in MCF10A normal breast epithelial cells) or (iii) increasing chromosome mis-segregation (using CENP-E inhibition in Cal51 breast cancer cells)).
The paper is in general well presented and holds interest for the readership of Plos Biology. I have a few concerns that would need to be addressed in order to strengthen the authors' current conclusions:

We thank the Reviewer for their thoughtful evaluation of our past and present work and for their questions and suggestions. In our view, addressing these comments has improved the manuscript.

Major points:

1. At present the manuscript seems to imply that the action of microtubule (MT) poisons in causing multipolar spindles is the cause of anti-tumour activity (presumably via cell death). For example, they state in the introduction "Timelapse analysis reveals that diverse microtubule poisons induce a mitotic spindle that starts out multipolar in early mitosis. In cases in which the multipolar spindle is sustained, it results in high CIN and cell death". However the data do not directly show this at present. In Figure 1 they do not correlate multipolar spindle incidence with subsequent cell death. In Figure 6 they use Cal51 cells and 5nM paclitaxel (stabiliser) or vinorelbine (destabiliser) and follow cell death rates, but there are a couple of issues with this data as it is currently presented. First, there seems to be a bit of a mismatch between the rates of multipolar spindles in Cal51 with these two agents between Figure 1 and 6. Cal51 only reaches about 50% multipolar spindles even at 10 nM in Figure 1, but in Figure 6 they show ~70% multipolar spindles in prometaphase at 5 nM paclitaxel for example. Can they comment on this? Or show the data for 5 nM in Figure 1 too?

The reason the multipolar spindle percentage is lower in Figure 1B than in Figure 6B “multipolar at prometaphase/metaphase” is because Figure 1B is the percentage of multipolar spindles in all stages of mitosis. Because Cal51 cells focus multipolar spindles as mitosis progresses, the percentage of multipolar spindles is higher in early stages of mitosis (~70% in prometaphase and metaphase, Figure 6B) and declines to ~35% at anaphase onset (Figure 6B). Some cells focus multipolar spindles after anaphase onset, further reducing the rate of multipolarity when quantifying anaphase and telophase cells. If combining all stages of mitosis in the timelapse analysis, spindle multipolarity is ~50%, similar to Figure 1B. The difference in the cell population analyzed is now clarified in the legend for Figure 1.

Second, they show the overall rates of cell death after paclitaxel and vinorelbine, but do not correlate with the incidence of the four categories of spindle multipolarity (2-2-2, etc). Instead, they analyse only the 3-2-2- category for cell death. They need to show the direct correlation between multipolarity (either transient or sustained) and cell death to support their statement in the introduction that ""Timelapse analysis reveals that diverse microtubule poisons induce a mitotic spindle that starts out multipolar in early mitosis. In cases in which the multipolar spindle is sustained, it results in high CIN and cell death".

We show sustained multipolarity (3+->3+->3+) corresponds with increased CIN and cell death in Figures 2F and 4B. We have also added Supplemental Figure 6, which shows that sustained multipolarity increases chromosome missegregation (Figure S6A-B) and that cells that maintain multipolar spindles longer are more likely to die (Figure S6A-D). Figure S6 also shows that this is based on the persistence of spindle multipolarity, not the type of microtubule poison used. This is discussed on page 11.

2. All the experiments showing sensitisation to multipolar spindle formation due to centrosome amplification are done in MCF10A cells, rather than in a cancer cell line with 'natural' centrosome amplification (CA). They only tested the synergy between MT poisons and CA using forced CA in normal breast epithelial cell line MCF10A. Why didn't they use a breast cancer line with CA to investigate this? They could have correlated the number of centrioles and proportion of time in prometaphase, anaphase with multipolar spindles etc with cell death. Modelling this in MCF10A normal cells I am not sure how they link this to patients? Do they propose that natural CA would synergise with taxol treatment? They could discuss this more.

The Reviewer raises an interesting point. We considered labeling centrosomes in MDA-MB-231 cells, which “naturally” have centrosome amplification (Acu et al, *Cancer Res* 2010; Mittal et al, *Sci Rep* 2017) and following these cells to determine whether cells with amplified centrosomes were less likely to focus multipolar spindles. However, the rate of focusing is so low in this cell line (Scribano et al, *Sci Transl Med* 2021, Fig 1G-H) that it hampered this analysis. We therefore introduced dox-inducible Plk4 into Cal51 cells,

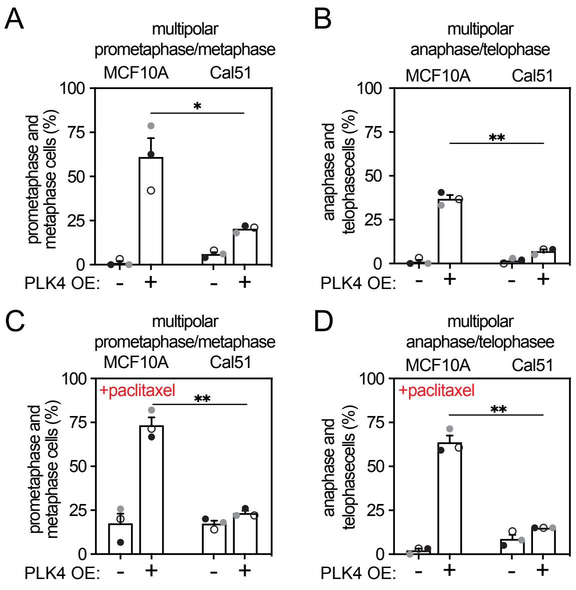

Figure for review. (A-B) Centriole amplification due to dox-inducible Plk4 overexpression induces multipolar spindle formation much more efficiently in MCF10A than Cal51 cells in early (A) and late (B) stages of mitosis. (C-D) Paclitaxel substantially increases the incidence of multipolar spindles in Plk4-expressing MCF10A cells but not Cal51 cells in early (C) and late (D) stages of mitosis. n ≥ 80 cells per condition across 3 biological replicates.

to determine whether this was sufficient to prevent the focusing that readily occurs in this cell line. Although dox-inducible expression of Plk4 produced centriole amplification in ~40% of Cal51 cells (new Supplemental Figure 5A-B), only ~20% of these cells had multipolar spindles in early stages of mitosis (prometaphase and metaphase; Fig. S5C) and <10% of spindles remained multipolar after anaphase onset (Fig. S5D). In comparison, though dox-inducible Plk4 expression caused a similar rate of centriole amplification in MCF10A cells (new Fig. S4C), in the absence of paclitaxel, Plk4 overexpression induced a substantially higher incidence of multipolar spindles in MCF10A cells than in Cal51 cells in early stages of mitosis (~60% versus ~20%; Figure for Review A). Similarly, Plk4 overexpression induced multipolarity in ~35% of MCF10A cells in late stages of mitosis but <10% of Cal51 cells (Figure for Review B). Paclitaxel treatment potentiated multipolar spindles in Plk4 overexpressing MCF10A cells (~75% of cells in early stages of mitosis and ~65% of cells in late mitosis), but not in Cal51 cells (Figure for Review C-D). These data demonstrate that centrosome amplification per se is not sufficient to predispose paclitaxel-treated cells to multipolar divisions, though it can do so in certain contexts. This is now discussed on page 9.

3. They present nice data carefully quantifying the levels of alteration in genomic content in daughter cells that originated after a 3-2-2 division, and conclude that loss of genomic material over 20% is highly likely to induce subsequent cell death. Both of the cell lines used to test this are diploid, or near diploid, whereas many cancer cell lines exhibit higher ploidy. MCF10A is a non-transformed breast epithelium line, and Cal51 is also a diploid triple negative cell line (very unusual). Therefore it is likely that most cancer cell lines would have to lose significantly more genomic material in order to be susceptible to nullisomy or monosomy. Either additional cell lines with higher ploidy more representative of breast cancer karyotypes should be tested in this assay, or their conclusions should be clearly caveated by explaining the genomic content of the cells used in the study. Also the authors need to state what is the p53 status of both these lines. P53 status isknown to be a large factor in whether diploid cells tolerate the gain, or loss of chromosomes. If they would like to propose that tumour cells could be sensitised to microtubule disrupting agents by increasing CIN then they would need to repeat experiments with more representative cancer cell lines that carry non-diploid karyotypes (and/or defective p53 signalling), to show that the proposed strategy of sensitising to MT agents by elevating CIN by a critical threshold of 3-4 chromosomes mis-segregating could be generally applicable to cancer treatment strategy. E.g. the MDA-MB-231 line is reported to have a triploid genome content which might be expected to easily buffer against the loss of 20% of the genome.

We thank the Reviewer for raising this critical point. Indeed, MCF10A and Cal51 cells are both near-diploid with wild type p53. We therefore analyzed timelapse analysis of MDA-MB-231 cells, which express the R280K mutant of p53, as suggested by the Reviewer. While we expected that the near triploid DNA content of MDA-MB-231 cells might buffer them against multipolar divisions, we found that loss of ≥20% DNA content resulted in death of ~80% of daughter cells (new Figure 4E), just as we had observed in MCF10A cells (Figure 4D). Loss of 6-19% of DNA content significantly increased cell death in MDA-MB-231 cells, as it

did in diploid MCF10A cells (Figure 4E). These data support our prior conclusion, but we realize that it is important to emphasize that the critical threshold is net loss of 3-4 chromosomes *in a diploid cell*, and that this would be proportionally higher in triploid and tetraploid cells (ie 4-6 chromosomes in a triploid cell). As chromosome missegregation results in chromosome gain as well as loss, the overall missegregation rate must likely be higher than 3-6 chromosomes in order to achieve net loss of this amount of DNA. These new data appear in Figure 4E, and are discussed on pages 11-12 and 19.

4. In discussion:"it is not possible at this point to determine whether the observed increase in cell death was due to nullisomy. Interestingly, previous work has indicated that chromosome missegregation is often non-random; in human cells, larger chromosomes tend to be preferentially missegregated [80- 83]. This may increase the likelihood of nullisomy after loss of a relatively small number of chromosomes". As far as I am aware no one has yet tested the identities of chromosomes mis-segregated more frequently after multipolar mitosis so it is not clear if larger chromosomes would be more highly mis-segregated in this situation. In my opinion, the more likely explanation is monosomy - which has been shown to be a high barrier to cell fitness, and difficult or impossible to obtain without p53 mutation (work from the Storchova lab). Could they comment on this more extensively in the discussion?

Good points! We have amended our discussion to specify that, though there is evidence that larger chromosomes tend to be missegregated, it is not yet known whether this is true after multipolar mitosis. We have also commented on the inability to achieve monosomic RPE1 cells in the presence of functional p53 (Chunduri et al, *Nat Commun* 2021) and the potential contribution of monosomy to cell death in our experiments (page 19).

5. In Figure 1 they detect increases in clinical tumour specimens of multipolar spindles after treatment with single agents. Their schematic indicates that the intratumoural concentration was also determined - can they show this data (sorry if I missed it somewhere) and discuss how this matched with the doses used in their experiments to monitor multipolar spindle formation?

We were exceedingly disappointed that the vinorelbine treated tumor cores were so small (<0.0005 mg) that the vinorelbine in those samples was below the lower limit of detection. We were able to measure the intratumoral concentration of eribulin in two tumor cores, which can be achieved by treatment with 5-10 nM eribulin in Cal51 and MDA-MB-231 cells. The schematic in Figure 1E and the legend for Figure 1 have been modified to indicate this. The intratumoral eribulin concentration is shown in the new Table S2.

Minor points:
1. Figure 1a: Why do they show images of cells treated with 5nM but that is not one of the concentrations they show data for?

We have changed the images in Figure 1A to show cells treated with 10 nM paclitaxel.

2. Figure 3 might be better as supplemental data?

Quantification of DNA content using histone H2B fluorescence is a novel method that, as Reviewers 1 and 2 indicate, will be useful for the field. We therefore consider validation of this method important to include as a main figure.

3. Figure 5. Legend could state cell type used

Thanks for bringing that to our attention. We have included the cell line used in the figure legend.

4. Sentence "Cell death occurred more rapidly in cells with ≥20% DNA loss than in cells that gained DNA, though even in these cells death often occurred relatively slowly, requiring >24 hours (Figure 4e)." is not very clear

Thanks for pointing this out. We made this into two sentences to improve clarity.

5. It is very striking and interesting that nocodazole does not cause this effect. Can they speculate in the discussion more on their thoughts on this?

This is a very interesting point, which your question prompted us to pursue further. We considered four factors that might be responsible for the differential ability of nocodazole to induce multipolar spindles: binding stoichiometry, reversibility of drug binding, ability to bind to soluble versus polymerized tubulin, and binding site. No single factor appears to be sufficient to account for the difference in inducing multipolarity. The difference does not appear to be due to binding stoichiometry, as substoichiometric concentrations of both nocodazole and clinically effective microtubule poisons suppress microtubule dynamics (Vasquez et al, *Mol Biol Cell* 1997, Jordan and Wilson, *Nat Rev Cancer* 2004, Derry et al, *Biochemistry* 1995). Nor is duration of drug binding to tubulin sufficient to explain the difference, as both nocodazole (Hoebeke et al, *Biochem Biophys Res Commun* 1976) and the vinca alkaloid vinblastine (Mukhtar et al, *Mol Cancer Ther* 2014) bind to tubulin in a rapidly reversible manner. Nor does the difference appear to be due to whether the drug binds both soluble tubulin and polymerized microtubules. Though taxanes bind only microtubule polymers (Derry et al, *Biochemistry* 1995), nocodazole, vinca alkaloids and eribulin bind to soluble tubulin subunits as well as microtubules (Lee et al, *Biochemistry* 1980; Jordan and Wilson, *Nat Rev Cancer* 2004; Smith et al, *Biochemistry* 2010). The difference is also not due exclusively to differences in the tubulin binding site. Taxanes binds along the length of the internal surface of polymerized microtubules rather than to soluble tubulin heterodimers. Epothilones bind to the same site and compete with paclitaxel for microtubule binding (Bollag et al, *Cancer Res* 1995, Kowalski et al, *J Biol Chem* 1997). Vinca alkaloids bind to the β subunit of tubulin dimers and to microtubule ends near the interface of the tubulin subunits. Eribulin binds to the vinca domain (Smith et al, *Biochemistry* 2010). Nocodazole, in contrast, binds to the colchicine binding site, which is located at the interface between the α and β subunits of the tubulin heterodimer (Hoebeke, J. et al., *Biochem Biophys Res Commun* 1976, Wang et al, *Drug Discov Today* 2022). However, colchicine has been reported to potently induce multipolar spindles at certain concentrations (Hervas et al, *Int J Cytology, Cytosystematics and Cytogenetics* 1974). Interestingly, colchicine binding to tubulin is poorly reversible (Herman et al, PNAS 1989; Jordan and Wilson, *Nat Rev Cancer* 2004), while nocodazole binding is rapidly reversible (Hoebeke et al, *Biochem Biophys Res Commun* 1976). This led us to test whether colcemid, which is structurally similar to colchicine and binds the same site on microtubules (Banerjee and Bhattacharyya, *FEBS Letters* 1979), but is rapidly reversible (Serpinskaya et al, *Biochim Biophys Acta* 1981, Frankel *PNAS* 1976) can induce multipolar spindles. Interestingly, it does not. Like nocodazole, treatment with increasing concentrations of colcemid causes the loss of mitotic spindles without a multipolar intermediate (Figure S3E-H). These data lead us to hypothesize that agents that bind in a rapidly reversible manner to the colchicine site of microtubules do not induce multipolar spindles. This is discussed on pages 6 and 17-18 of the revised manuscript.

6. Would be easier to understand and compare the data if S1 and S2 were merged (as they are in S3).

We considered this. However, Figure S1 displays data for 4 drugs while S2 has data for 7 drugs. Additionally, though high concentrations of microtubule destabilizers cause the maximum mitotic index, we are not able to quantify spindle polarity at these high concentrations because they completely depolymerize microtubules. We therefore kept Figures S1 and S2 separate.

7. A small box listing the drugs and whether they are an MT stabilisier or destabiliser would be helpful in Figure 1.

Thanks for this input. We've added this to Figure 1 as panel D (and also Figures S1 and S2).

8. Figure 2 - why is no significance shown compared to DMSO? Data looks significant. Is it just because box is getting "too busy" with too many stars?

Yes, we did not include certain statistical comparisons out of concern that the graph would look too busy. However, this question was also raised by Reviewer 2, and we have now included the requested comparisons.

9. Graphs 2G/H/I feel redundant. Could this be streamlined? The authors comment that it is interesting that cell death takes 34hrs. Why is that interesting? Can they expand on that.

Without panels 2G and 2I, the data in panels 2F and 2H make a strong case that prolonging the persistence of multipolar spindles increases cell death. However, they leave open the question of whether this varies based on the microtubule poison that contributes to spindle multipolarity. Panels 2G and 2I are necessary to show that the effect is due to the type of division, and that both microtubule poisons have a similar mechanism. Panel 2H highlights a key feature that is not often taken into account when examining the consequences of mitotic defects – time. The fact that cells take, on average, ~34 hours to die following substantial DNA loss caused by multipolar divisions is surprising, as apoptosis can occur within as little as 10 minutes following mitochondrial release of cytochrome c and the activation of caspases (Green, *Cell* 2005). The delayed and highly variable timing of cell death emphasizes the need for long term timelapse analysis to accurately assess cell death following abnormal mitosis but also suggests that the cause, and perhaps the mechanism, of cell death is variable and depends on the karyotype of the daughter cell rather than the mere state of lethal DNA loss. As noted earlier, experimental induction of any monosomy is not tolerated in p53-competent RPE1 cells (Chunduri et al, Nat Commun 2021) and could be predicted to give rise to cell death with similar timing in p53 competent MCF10A cells. Similarly, if there were a general cellular response to nullisomy, this could be expected to cause relatively rapid cell death with a similar time course. However, this is not observed. This topic is now discussed on page 17.

10. Figure 3: the authors said the DNA contents are in the "expected range." It seems to be roughly 3-4%. Can they clarify what they expected and how they calculate this in the text.

The expected range was calculated as a percentage of the chromosome length in base pairs relative to the sum of base pairs across all chromosomes in mitosis. In a female diploid system, the largest and smallest chromosomes, chromosomes 1 and 21, make up ~4.1% and ~0.8% of the genome, respectively. This is now included in the legend for Figure 3.
